# Antimigratory Effects of the Methanol Extract from *Momordica charantia* on Human Lung Adenocarcinoma CL1 Cells

**DOI:** 10.1155/2012/819632

**Published:** 2012-12-18

**Authors:** Hsue-Yin Hsu, Jung-Hsuan Lin, Chia-Jung Li, Shih-Fang Tsang, Chun-Hao Tsai, Jong-Ho Chyuan, Shu-Jun Chiu, Shuang-En Chuang

**Affiliations:** ^1^Department of Life Sciences, Tzu-Chi University, Hualien, Taiwan; ^2^Institute of Medical Sciences, Tzu-Chi University, Hualien, Taiwan; ^3^Department of Anatomy, Tzu-Chi University, Hualien, Taiwan; ^4^Hualien District Agricultural Research and Extension Station, Hualien, Taiwan; ^5^National Institute of Cancer Research, National Health Research Institutes, Zhunan, Taiwan

## Abstract

*Momordica charantia* has been found to exhibit anticancer activity, in addition to its well-known therapeutic functions. We have demonstrated that the leaf extract of *Momordica charantia* (MCME) induces apoptosis in several human cancer cells through caspase- and mitochondria-dependent pathways. In this study, a different susceptibility to MCME was found in human lung adenocarcinoma CL1 cells with different metastatic ability, leading to the significant difference of cell viability and invasiveness between MCME-treated CL1-0 and CL1-5 cells. MCME was found to upregulate the expression of Wnt-2 and affect the migratory and invasive ability of CL1 cells through suppressed MMP-2 and MMP-9 enzymatic activities. We proposed that MCME mediates inhibition against migration of CL1 cells by reducing the expression and activation of Src and FAK to decrease the expression of downstream Akt, **β**-catenin, and MMPs.

## 1. Introduction

Lung cancer is the most prevalent cancer and has become the number one cancer cause of death in some countries. The major health threat by lung cancer is metastasis through a multistep process [1]. During the tumor progression, cancer cells can escape control of their surrounding microenvironment, invade and penetrate the walls of lymphatic or blood vessels, and eventually migrate to distant sites within the body [2]. Critical characteristics that cancer cells have acquired for metastasis include the ability to penetrate the basement membrane and degrade the extracellular matrix (ECM) which is mediated mainly by matrix metalloproteinases (MMPs). Focal adhesion kinase (FAK), a cytoplasmic kinase that is involved in ECM/integrin-mediated signaling pathways, is overexpressed in a variety of cancers and has been suggested to modulate the migration and invasion of tumor cells [3, 4]. FAK localizes to focal contact sites and has been linked to the generation of cell survival, cell cycle progression, and cell motility, in part through the recruitment of Src and other adaptor proteins into a focal contact-associated signaling complex [5]. Src becomes activated in response to cell-cell adhesion and is activated in most invasive cancers [6]. Upon activation, Src phosphorylates FAK which is required for cell adhesion and cell spreading [6, 7]. Activation of the functionally interdependent protein kinase cascade, including Src and FAK, has been suggested to play the key role on cell migration and invasion [8]. Metastasis of lung cancer was reported to be occurred by MMPs through the regulation of FAK [1, 9]. Increasing evidence suggests that the Wnt signaling pathway is involved in tumor development and/or progression [10]. Wnt-2 overexpression is thought to be involved in human lung carcinogenesis and inhibition of Wnt-2-mediated signaling induces programmed cell death in non-small-cell lung cancer cells [11]. The canonical Wnt pathway modulates glycogen synthase kinase 3*β* (GSK-3*β*) and *β*-catenin activity by regulating the cellular localization through a cascade of events that result in nuclear localization of *β*-catenin and activation of transcription by interacting with transcription factors [12, 13].

The invasion and metastasis of tumor cells are characteristics of highly malignant cancers with poor clinical outcome and considered to be the most difficult problems for cancer therapy. Unlike that in the non-small-cell lung cancer, widespread metastasis in lung adenocarcinoma occurs relatively late [14]. It is clinically more effective for treating lung adenocarcinoma by forbidding the development of invasiveness as early as possible. Therefore, complementary strategies with nonsurgical treatment may be needed to prevent lung cancer progression and improve patient survival rates. 

*Momordica charantia* (MC), also known as bitter gourd, is extensively used as a vegetable in tropical areas. A number of *in vitro* and *in vivo* studies have found that MC possesses antidiabetic [15], abortifacient [16], anthelmintic [17], and contraceptive [18] effects. In addition, several studies have demonstrated that leaf or fruit extracts of MC exert antineoplastic effects against various cancers. Extracts of MC have been shown to inhibit proliferation of human breast cancer cells by inducing cell cycle arrest and apoptosis [19]. Leaf extracts inhibited P-glycoprotein-mediated drug efflux, resulting in an increase in the efficacy of chemotherapeutic drugs in multidrug-resistant human cervical KB-V1 carcinoma cells [20]. They have also been reported to prevent the secretion of matrix metalloproteinases (MMPs) and inhibit cell migration in a rat prostate cancer cell line [21]. Bioactive properties of MC against numerous cancers were demonstrated to be contributed by compounds with anticancer potential. Phytochemicals in MC that have been documented with cytotoxicity on cancer cells include proteins, triterpenoids, and their glycosides [22]. Ribosome-inactivated proteins and a chemical analogue, MAP30, in MC have been reported to exhibit the cytotoxicity and inhibit the metastasis of the highly metastatic human breast cancer MDA-MB-231 cells and considered to be potential therapeutic agents against breast carcinomas [23, 24]. In a previous study, we demonstrated the apoptosis induced by methanol extract of MC (MCME) on human lung adenocarcinoma CL1-0 cells through caspase- and mitochondria-dependent pathways, which changes of the antiproapoptotic Bcl-2 and proapoptotic Bax proteins were included [25]. We have tested the cytotoxicity of MC extracts on a series of human lung adenocarcinoma CL1 cells and found that the susceptibility of CL1 cells to MCME depends on their invasive ability. Here, the effect of MCME on CL1 cells is not only evaluated by cell viability, but also that of migration and invasion, in an attempt to characterize the mechanisms involved in MCME-reduced metastasis in lung cancer via comparing CL1-0 and CL1-5 cells, with distinct invasive ability individually. 

## 2. Materials and Methods

### 2.1. Chemicals and Antibodies

DMEM medium, RPMI-1640 medium, 3-(4,5-Dimethyl-thiazol-2-yl)-2,5-diphenyltetrazolium bromide (MTT), Trypsin-EDTA, penicillin/streptomycin, protease inhibitors, dimethyl sulfoxide (DMSO), EDTA, gelatin, crystal violet, SDS, Triton X-100, Tris, Tween-20, CaCl_2_, NaCl, NaN_3_, acetic acid, methanol, and all other miscellaneous chemicals used in this study were purchased from Sigma Chemical Co. (St. Loius, MO, USA). The antibody against MMP-2 (GTX104577), MMP-9 (GTX100458), Src (GTX63364), phospho-Src (GTX50210), FAK (GTX100764), phospho-FAK (GTX24803), PI3K (GTX111173), Akt (GTX13990), Wnt-2 (GTX62603), GSK-3*β* (GTX59752), phospho-GSK-3*β* (GTX59576), Vimentin (GTX100619), *β*-catenin (GTX101435), *β*-actin (GTX11003), and HRP conjugated goat anti-mouse or anti-rabbit IgG were all purchased from Genetex Inc. (ICON-GeneTex, Taipei, Taiwan). 

### 2.2. Preparation of MC Methanol Extract

MC cultivated in the Hualien agriculture research and extension station (HARES, Hualein, Taiwan) with introgressed characteristics between cultivars and wild species was identified and deposited as a voucher specimen, number 2381. Leaves of the authenticated MC were dried at 35 ~ 40°C and soaked in methanol at room temperature for two months after harvesting from HARES. After that, extract of methanol was filtered and centrifuged at 500 ×g for 10 min to remove precipitates and concentrated by rotary evaporation. The extracted residue was subsequently lyophilized at −80°C and then kept at −20°C until required for treatment. Methanol extract of MC (MCME) was dissolved in DMSO with a stock concentration of 200 mg/mL before dilution with media. 

### 2.3. Cell Culture

Human lung adenocarcinoma cell lines CL1-0, CL1-5 and A549 were used with two human embryonic cell lines, HEK293 and WI-38 cells, as normal control cells in this study. CL1 cells were originally established from a single cell clone isolated from the lung adenocarcinoma tissues of a patient with a poorly differentiated adenocarcinoma [24]. CL1-0 and CL1-5 cells, CL1 series of cell lines with distinct invasive and metastatic capacities [13], were kindly provided by Dr. S. E. Chuang (National Institute of Cancer Research, National Health Research Institutes, Taiwan). A549 cells were used to compare the susceptibility of CL1 cells to MCME in this study. CL1-0 and CL1-5 cells were maintained in RPMI-1640 medium, whereas DMEM medium was used for maintaining A549 cells, HEK293 cells, and WI-38 cells, supplemented with 10% fetal bovine serum, 0.1 mg/mL of streptomycin, and 100 units/mL of penicillin. All cells were incubated at 37°C in a humidified atmosphere containing 5% CO_2_. 

### 2.4. MTT Assay

MTT assay is a colorimetric assay based on the ability of viable cells to change from soluble yellow tetrazolium salt to blue formazan crystals. Cell lines evaluated in this study were maintained, dislodged, and suspended in each appropriate medium. An appropriate number of cells were added to 96-well plates and cultured for 24 h to become nearly confluent. After treating with MCME for 24 h, MTT was freshly prepared and incubated with cells at a concentration of 0.5 mg/mL for 4 h. The formazan crystals were dissolved in 100 *μ*L of DMSO and the optical density was read by an ELISA reader (Bio-tek Instruments, VT, USA) at 570 nm. Each treatment was tested eight times in at least three independent plates. Cell viability of cells treated with MCME was determined by comparing the absorbency of the untreated cells.

### 2.5. Cell Migration and Invasion Assays

Migration and invasion were determined using the wound healing and transwell assays by CL1-0 and CL1-5 cells, respectively. For determining the cell motility, equal number of CL1-0 and CL1-5 cells (5 × 10^5^/well) were seeded and grown overnight to 90–95% confluence in 12-well plates. Migration was tested in wound-healing assays using culture inserts (ibidi, Martinsried, Germany). Cellular debris was removed with phosphate buffer saline and cells were cultured with medium containing 0.15, 0.3, 0.6, and 1.25 mg/mL MCME. Wound closure was evaluated and photographed at 24 h with an inverted microscope (Olympus IX71 fluorescence microscope, Melville, NY, USA).

Cell invasion assays were performed using transwell chambers (Becton Dickinson, Franklin Lakes, NJ, USA). Cells (1 × 10^4^ cells/mL) were placed in the upper chamber which was filled with RPMI-1640 medium or with medium supplemented with 0.15, 0.3, 0.6, and 1.25 mg/mL MCME, while RPMI-1640 supplemented with 10% FBS was added in the bottom chamber. Incubation was carried out at 37°C for 24 h. The polycarbonate filters with 8 *μ*m pores were removed and cells on the upper filter surface were wiped away with a cotton swab. The filters were subsequently fixed with 100% methanol for 8–10 min at room temperature, stained with 0.2% w/v crystal violet, washed with PBS (pH = 7.4), and counted under a light microscope operating at 200x magnification. The invasion index was defined as the ratio of the percent invasion obtained with invaded CL1-0 or CL1-5 cells to the percent invaded obtained with noninvaded cells.

### 2.6. Gelatin Zymography

CL1-0 and CL1-5 cells were starved for 24 h with serum-free medium. Subsequently, cells in media containing 0.5% FBS were treated with 1.25 mg/mL MCME for different time periods, and thereafter, the supernatants were collected. The samples were analyzed with gelatin zymography (0.1% w/v) to assess the enzymatic activity of MMPs by using gelatin as the substrate. Each lane was loaded with a total protein concentration of 3 *μ*g and subjected to SDS-PAGE electrophoresis at 48°C. Gels were washed twice in 50 mM Tris (pH = 7.4) containing 2.5% (v/v) Triton X-100 for 1 h, followed by repeated 10-min rinses in 50 mM Tris (pH = 7.4). Gels were then incubated overnight in 50 mM Tris (pH = 7.5) containing 10 mM CaCl_2_, 0.15 M NaCl, 0.1% (v/v) Triton X-100, and 1% NaN_3_ at 37°C with gentle shaking overnight. After incubation, gels were stained with 0.25% Coomassie brilliant blue and destained in 7.5% acetic acid with 20% methanol. Matrix metalloproteinases in the loaded supernatants leads to the gelatinase bands appearing white on a blue background.

### 2.7. Western Blot Analysis

CL1-0 and CL1-5 cells were harvested after treatment with 1.25 mg/mL MCME for varying intervals. Cells were lysed with buffer containing 1% Triton X-100, 50 mM Tris (pH = 7.5), 10 mM EDTA, 0.02% NaN_3_, and a protease inhibitor mixture. Proteins were collected and determined by Bradford protein assay kit (Amresco, OH, USA) before boiling with the electrophoresis sample buffer for 5 min. Equal protein content of treated cells were resolved in 12% SDS-PAGE gels. Proteins were then blotted on a nitrocellulose membrane (Millipore Co., MA, USA) and blocked for 1 h in blocking solution (5% nonfat dry milk, 1% Tween-20 in PBS) at room temperature. Membranes were washed with Tris-buffer saline (with 0.1% Tween-20) and probed with specific primary antibodies for 1 h at room temperature. After washes with TBS-T, blots were incubated with a 1/5000 dilution of HRP conjugated goat anti-mouse or anti-rabbit IgG for 1 h at room temperature. Protein bands were developed by the Immobilon Western Chemiluminescent HRP Substrate (Millipore Co.) and observed under UVP Bioimaging systems (UVP Inc., Upland, CA, USA).

### 2.8. Statistical Analysis

Quantification of all experiments was conducted using a densitometer (Personal Densitometer SI, Molecular Dynamics, CA, USA) for statistical analyses. Data presented are the mean ± SD. from at least three independent experiments. In all experiments, significance of difference between two groups was calculated by a Tukey's test after one-way analysis of variance (ANOVA) by SPSS 13.0 software.

## 3. Results

### 3.1. Cytotoxicity of MCME to Human Lung Adenocarcinoma Cells

**Figure 1:**
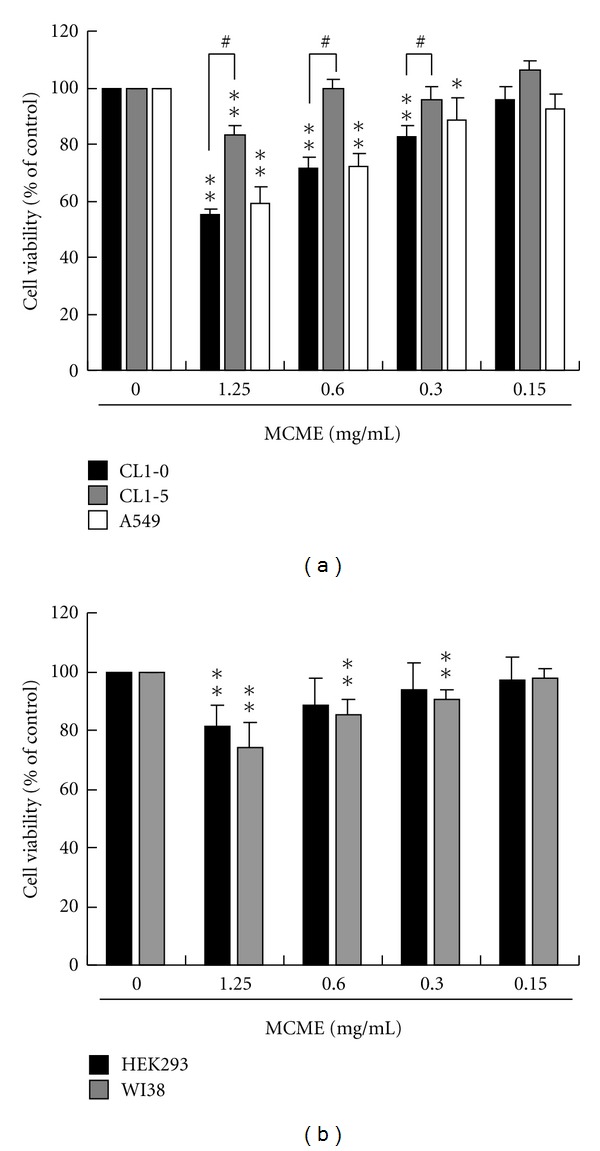
Effect of MCME on lung cancer and normal epithelial cells. (a) CL1-0, CL1-5, and A549 cells were treated with MCME for 24 h at concentrations ranged from 0.15 to 1.25 mg/mL. (b) HEK293 and WI-38 cells were treated with MCME at the same condition as that in (a). After treatment, cell viability was determined using the MTT assay. Data derived from at least three independent experiments, eight tests for each, were represented by mean ± SEM. * and ** indicate *P* < 0.05 and 0.01, respectively. ^#^ indicates the difference between CL1-0 and CL1-5 cells is significant (*P* < 0.05).

The cytotoxicity of MCME on three human lung adenocarcinoma cell lines, CL1-0, CL1-5, and A549, was evaluated by the MTT assay. As shown in Figure 1(a), cells treated with varying concentrations of MCME (0.15, 0.3, 0.6, and 1.25 mg/mL) for 24 h resulted in a significant reduction in cell proliferation of CL1-0 and A549 cells, whereas a distinct susceptibility of CL1-5 was found. The reductions in viability for each cell line after exposure to MCME for 24 h were 55.2 ~ 95.8% for CL1-0 cells, 59.2 ~ 92.6% for A549 cells, and 83.3 ~ 105.8% for CL1-5 cells. These results indicated that MCME inhibited the proliferation of A549 and CL1-0 cells in a dose-dependent manner, with the most potent antiproliferative effect against CL1-0 cells but not the highly invasive CL1-5 cells [14]. As shown in Figure 1(b), cell viability of MCME-treated normal cells indicated that cytotoxicity of MCME on normal human embryonic kidney HEK293 cells and lung WI-38 cells was significant at 1.25 mg/mL, while it was not at 0.15 mg/mL. HEK293 cells represented a less susceptibility to MCME than that of WI-38 cells. Hence, viability of MCME-treated normal cells was not reduced as much as that of CL1 and A549 cells. The distinct antiproliferative effect of MCME at 24 h between CL1-0 and CL1-5 cells indicated that MCME-induced different cytotoxicity in CL1 cells. The differential cytotoxicity between human normal cells and human lung adenocarcinoma cells demonstrates the ascendant to use MCME as a remedy in cancer prevention. Therefore, CL1-0 and CL1-5 cells were subsequently chosen in investigating the antimetastatic effect of MCME on lung adenocarcinoma cells.

### 3.2. Effect of MCME on the Migration of CL1 Cells

As shown in Figure 1(a), cell viability of CL1-0 and CL1-5 cells represented significant difference to MCME at 0.3, 0.6, and 1.25 mg/mL. We selected 1.25 mg/mL, a concentration reduced about 45% and 17% cell viability at 24 h for CL1-0 and CL1-5, respectively, as the highest concentration to further assess the effect of MCME on the cellular migration and invasive properties.

**Figure 2:**
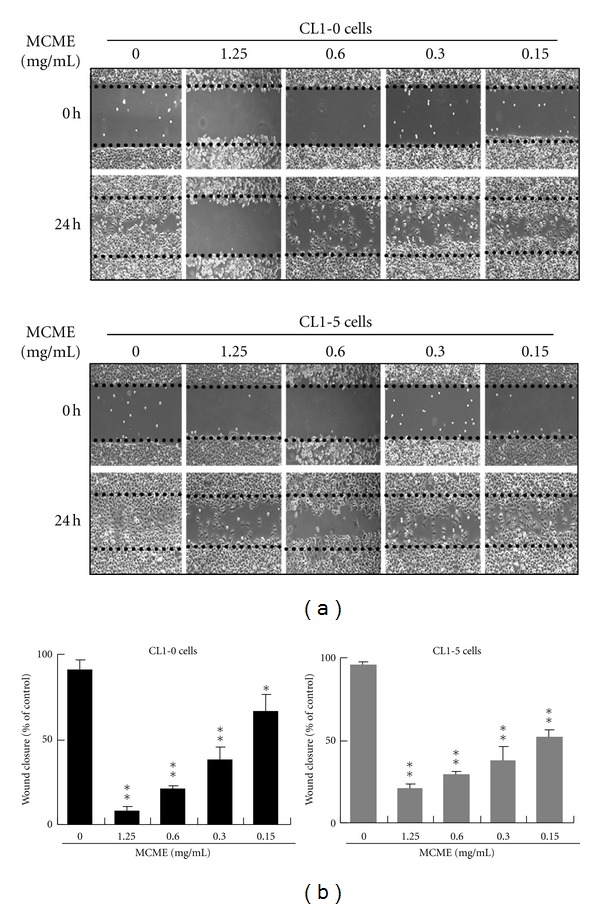
Effects of MCME on the migration of CL1-0 and CL1-5 cells. (a) Representative photographs from three independent experiments showing a dose-dependent inhibition of migration by treating with MCME for 24 h. Images of wound closures were captured using a microscope with a 10x objective. (b) The black dotted lines indicate the wound edge. Cell-free areas invaded by migrated cells across the black dotted lines were calculated by three randomized fields for each treatment and quantified as shown in the lower panels. Data derived from three independent experiments were represented by mean ± SEM. * and ** indicate *P* < 0.05 and 0.01, respectively.

Wound closure was examined at 24 h following treatment. As shown in Figure 2(a), control cells migrated into the wound and filled with the area at 24 h, especially for that of CL1-5 cells. The migration of MCME-treated CL1-0 cells was almost inhibited at concentration of 1.25 mg/mL. Wound closure was shown to be inhibited by MCME treated at 0.15 mg/mL, a concentration without affecting the cell viability, on both CL1-0 and CL1-5 cells. Quantified data indicated that MCME inhibited the motility of CL1-0 and CL1-5 cells in a dose-dependent manner, with 91.8% and 78.7% reductions at 1.25 mg/mL after incubating for 24 h, respectively (Figure 2(b)).

### 3.3. Effect of MCME on the Invasion of CL1 Cells

**Figure 3:**
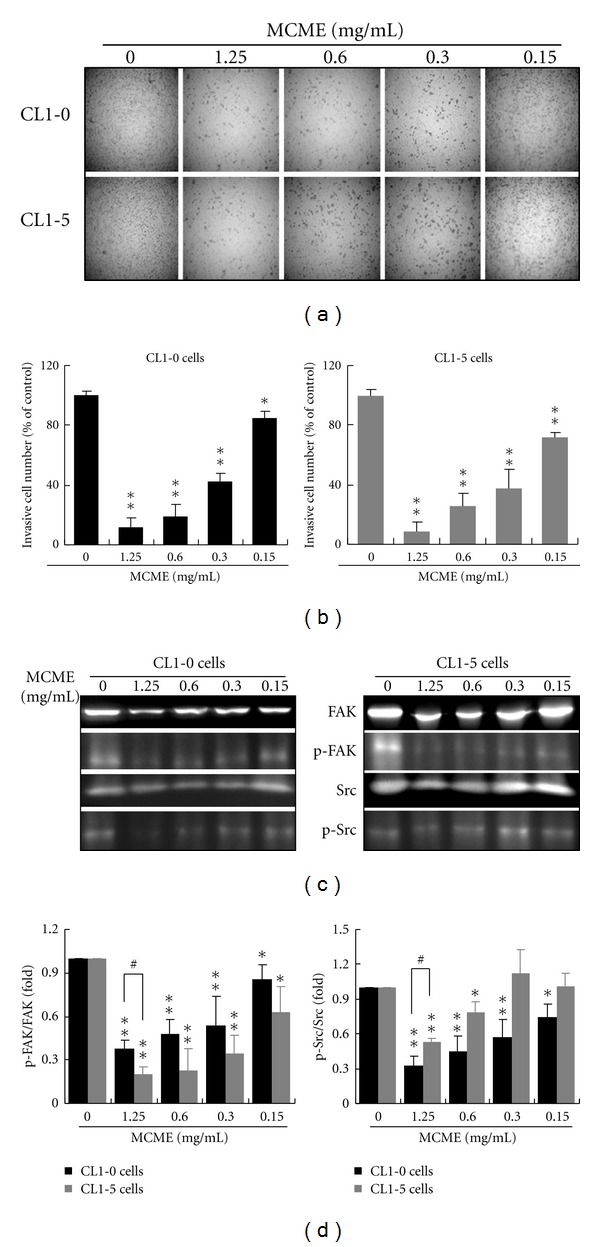
Effects of MCME on the invasion of CL1-0 and CL1-5 cells. (a) Invasiveness of CL1 cells treated with MCME. MCME induced a dose-dependent inhibition of invasion in CL1 cells. CL1-0 and CL1-5 cells were pretreated with MCME or DMSO (vehicle control) for 24 h and then seeded onto the transwell chamber. Photographs were taken with an inverted microscope with 10x magnification. (b) The invasive abilities of CL1 cells were quantified by enumerating the stained cells that invaded into the underside of the porous polycarbonate membrane. (c) Expression and phosphorylation of Src and FAK in CL1-0 and CL1-5 cells after treatment with MCME at 0 ~ 1.25 mg/mL for 24 h. (d) The protein activities were normalized to *β*-actin and expressed as the fold change to the respective control (0 h). Data derived from three independent experiments were represented by mean ± SEM. * and ** indicate *P* < 0.05 and 0.01, respectively. ^#^ indicates the difference between CL1-0 and CL1-5 cells is significant (*P* < 0.05).

CL1-0 and CL1-5 cells treated with MCME at 1.25 mg/mL for 24 h resulted in a significant decrease of invasion as compared to the untreated cells (Figure 3(a)). A similar result was shown in cells treated with lower concentrations of MCME. Invasion of CL1-0 and CL1-5 cells was prohibited by MCME in a dose-dependent manner, even at concentrations without significant cytotoxicity on CL1-5 cells. Quantified data indicated that MCME at concentrations from 0.15 to 1.25 mg/mL obviously decreased the invasion cells on both CL1 cells (Figure 3(b)). Expression of Src and FAK was decreased in both cells treated with MCME at 1.25 and 0.6 mg/mL (Figure 3(c)). As shown in Figure 3(d), MCME inhibited the activation of FAK and Src proteins of CL1-0 cells in a dose-dependent manner, whereas it was only found by the activation of FAK in CL1-5 cells. Inhibition of Src activation was induced by MCME treated at 0.6 and 1.25 mg/mL in CL1-5 cells. MCME-inhibited activation of Src and FAK was significantly different between CL1-0 and CL1-5 cells at 1.25 mg/mL.

### 3.4. Effect of MCME on the Expression and Activity of MMPs

**Figure 4:**
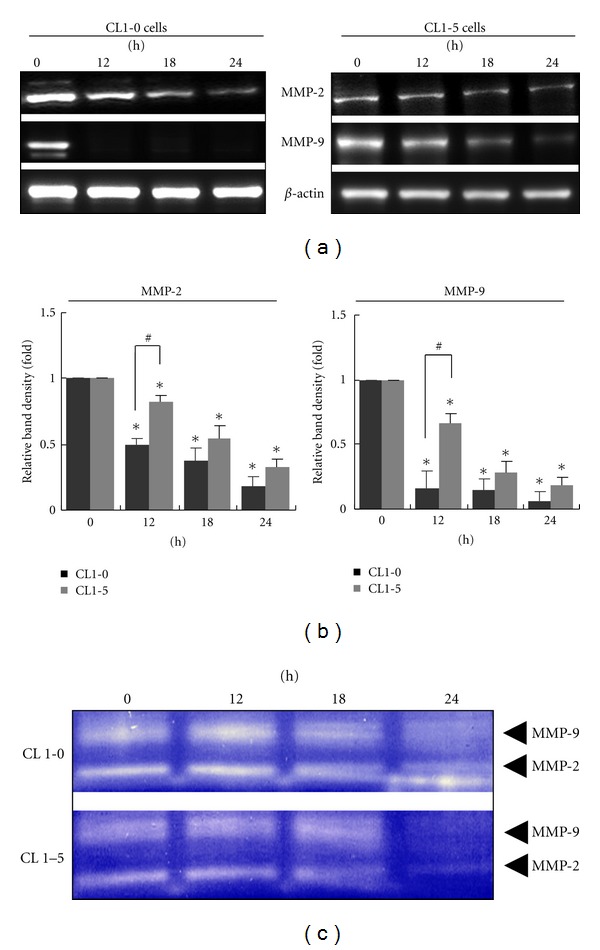
MMPs activities in MCME-treated CL1-0 and CL1-5 cells. (a) Expression of MMP2 and MMP9 in MCME-treated CL1-0 and CL1-5 cells. Expression of MMP-2 and MMP-9 was decreased after treating with MCME at 1.25 mg/mL. (b) Quantified expression of proteins in MCME-treated CL1 cells. The protein expression levels were normalized to *β*-actin and expressed as the fold-change to the respective control (0 h). Data derived from three independent experiments were represented by mean ± SEM. * indicates a significant difference of *P* < 0.01 as compared with control groups, whereas ^#^ indicates *P* < 0.05 between two groups. (c) The enzymatic activities of MMP-2 and MMP-9 of CL1-0 and CL1-5 cells treated with MCME at 1.25 mg/mL. The MMP activities in cultured media were determined by gelatin zymography and identified by clear zones of digested gelatin.

To elucidate the MCME-mediated inhibitory effect of cell migration on CL1-0 and CL1-5 cells, expression and activity of MMP2 and MMP9 were determined. As shown in Figure 4(a), MMP-2 and MMP-9 protein levels were decreased gradually in MCME treated CL1-5 cells, while MMP-9 in CL1-0 cells was significantly reduced from 12 h to 24 h (Figure 4(b)). The cultured media of cells treated with MCME were collected and the MMP activity was measured by gelatin zymography. Gelatin zymography assays showed that both cell lines treated with MCME exhibited decreased activities of MMP-2 and MMP-9. As shown in Figure 4(c), MMP-2 and MMP-9 activity was decreased significantly at 24 h in the CL1 cells, indicating a potent inhibition of MMPs was induced by MCME in CL1-0 and CL1-5 cells.

### 3.5. MCME Affects the PI3K/Akt/GSK-3 and Wnt Pathways in CL1 Cells

**Figure 5:**
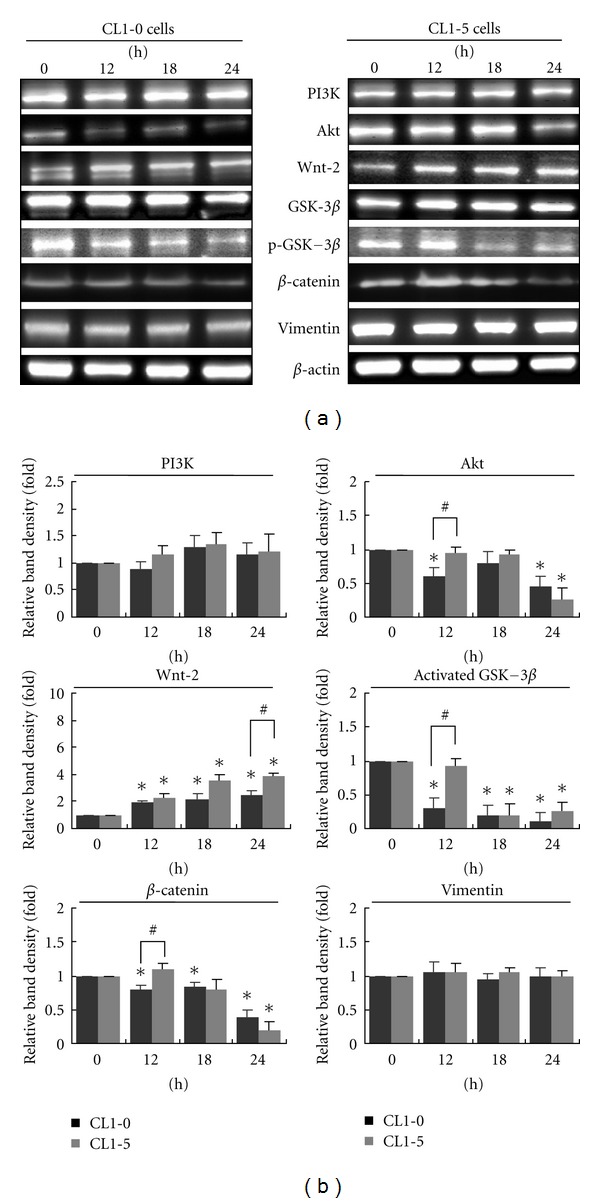
Effects of MCME on migration-related proteins in CL1-0 and CL1-5 cells. (a) Cells were collected at the indicated time points after treatment with 1.25 mg/mL MCME. Time-dependent effects of MCME on the protein expression level of PI3K, Akt, Wnt-2, GSK-3*β*, phosphorylated GSK-3*β*, *β*-catenin, and vimentin in CL1-0 and CL1-5 cells were evaluated by western blot. (b) The protein expression levels were normalized to *β*-actin and expressed as the fold-change to the respective control. Each value represented by the mean ± SEM. is quantified by representative blots from 3 independent experiments. * indicates a significant difference of *P* < 0.01 as compared with control groups, whereas ^#^ indicates *P* < 0.05 between two groups.

The PI3K/Akt pathway promotes cell survival by both enhancing the expression of antiapoptotic proteins and inhibiting the activity of proapoptotic ones. Opposite to the inactivation by Wnt proteins, glycogen synthase kinase 3 (GSK-3) is phosphorylated by Akt at Ser9 [26]. Inactivated GSK-3 retards the specific targeting of protein expressions for cell proliferation or metastasis [27]. As shown in Figure 5, the expressions of Akt were dramatically decreased in CL1-0 cells at 12 h, whereas CL1-5 cells demonstrated a slower reduction of Akt level after MCME treatment, reaching their lowest level at 24 h. Expressions of PI3K were constant and the Wnt-2 proteins were increased from 12 h in both cell lines. GSK-3*β* phosphorylated at Ser9 followed a similar time dependency as Akt expression. However, expression of GSK-3*β* was decreased in CL1-0 cells, while it was increased in CL1-5 cells, at 24 h after treatment by MCME. Opposite to the increased expression of Wnt-2 in both CL1 cells, expression of *β*-catenin was decreased by MCME treatment for 24 h. Vimentin, which is known to be overexpressed in lung cancer and correlated well with accelerated tumor growth and invasion [27], remained unchanged in MCME-treated CL1 cells. These data clearly demonstrated that Akt, Wnt, GSK-3*β*, phosphorylated GSK-3*β*, and *β*-catenin were involved in the MCME induced cytotoxicity and antimigration activity.

## 4. Discussion

Tumors commonly defined as malignant reflect a higher ability of cells to proliferate and invade into surrounding tissues that may be caused by growth pressure or the cellular capability of migration [28]. Migration and invasion were reported to be key biological features of lung adenocarcinoma cells [29]. CL1 cells were reported to be tumorigenic but not highly metastatic unless cultured with passages and a series of heterogeneous sublines with progressive invasiveness were cloned. Among cell lines used in this study, CL1-5 cells as well as A549 cells were reported to exhibit the highly invasive and metastatic potentials [9, 14]. However, our results indicated that CL1-0 and A549 exhibit more susceptible to MCME than CL1-5 cells at concentrations ranged from 0.15 to 1.25 mg/mL. In addition, MCME at a concentration even without significant cytotoxicity on normal kidney and lung cells resulted in the delayed migration of CL1-0 and CL1-5 cells. The antimetastatic effect of MCME in both CL1-0 and CL1-5 cells was dose dependent, suggesting an effect other than antiproliferation dominated the role of MCME in CL1 cells. 

FAK was reported to promote lung cancer cell migration through the MAPK signaling and MMPs pathway [1, 30]. Several FAK downstream signaling pathways have been implicated in mediating FAK promotion of cell migration. One of the particularly well characterized pathways is through the association and phosphorylation of adaptor molecules by the Src/FAK complex [31]. FAK binds to PI3K, leading to increased activation of adaptor molecules or downstream effectors, which is another pathway mediating FAK promotion of cell migration [32]. Moreover, FAK promotes the expression of MMP2 and 9 through the Src-mediated signaling cascade and induces activation of cell survival signals as well as the secretion of MMPs into the matrix in cancer cells [33]. FAK, as a critical factor for developmental and pathological angiogenesis, was found to form a signaling complex in a Src-dependent manner, which is essential for angiogenic responses to tumor metastasis [34, 35]. Indeed, control of FAK signaling has been suggested as a potential anticancer and antimetastasis strategy [36]. Interestingly, we found *M. charantia* exhibiting anticancer as well as anti-angiogenesis in a mouse model with breast cancer MDA-MB-231 cells (Supplementary material available online at doi:10.1155/2012/819632). In this study, MCME-inhibited expression and phosphorylation of Src and FAK were found in CL1-0, while they were not for Src in CL1-5 cells at 0.15 and 0.3 mg/mL in which MCME-induced cytotoxicity was not significant. In contrast to cell viability, the suppressed migration and invasiveness in CL cells as shown at lower concentrations were independent of MCME-induced cell death. To further clarify the antimetastatic effect of MCME on CL1-0 and CL1-5 cells, we comparatively investigated some motility factors governing the metastasis. Metalloproteinases such as MMP-2 and MMP-9 are degradative enzymes that play critical roles in the invasion [37]. They are highly expressed and correlated with tumor aggressiveness and invasiveness as well as poor clinical prognosis in lung adenocarcinoma [38, 39]. A differential expression of MMPs, especially for MMP-9, in CL1-5 cells was known to regulate the higher invasive ability than that of CL1-0 cells [14]. To clarify the changes of MMPs induced specifically by MCME, we quantified and evaluated the MMP-2 and MMP-9 in CL1 cells individually. In addition to that treated with MCME for 12 h, expression of MMP-9 was not found to be significantly different between CL1-0 and CL1-5 cells. Thus, the inhibition of MMP activity is important for determining the ability of antimetastasis by MCME on CL1 cells. Suppression of activated Src and FAK coincided with the decreased expression of MMP-2 and MMP-9, suggesting that MCME-inhibited migration and invasion were mediated through Src/FAK/MMPs cascade in both CL1-0 and CL1-5 cells. While Src was not significantly inactivated in MCME-treated CL1-5 cells at lower concentrations, it indicated the inhibition of invasive ability by MCME was mediated by other FAK pathways. 

An increased level of *β*-catenin accompanied by the augmented MMP-9 expression is currently recognized as a potent mitogenic signaling molecule that leads to *β*-catenin-driven cell proliferation [37]. MCME suppressed MMP-2 and MMP-9 expressions but did not significantly influence expression of *β*-catenin in CL1-5 cells until 24 h after treatment, indicating the importance of *β*-catenin stability for cell proliferation. In contrast to the cell viability of MCME-treated CL1 cells, PI3K/Akt signaling pathway, which is another pathway mediating FAK promotion of cell migration as well as the mitogenic process of cancer cells, is included in this study. PI3K catalyzes the production of phosphatidylinositol 3, 4, 5-triphosphate (PIP3), a second messenger that is essential for Akt signaling at the plasma membrane [40]. Akt is a serine/threonine kinase that functionally modulates numerous substrates involved in the regulation of cell proliferation/survival, angiogenesis, and tissue invasion [41]. Akt is overexpressed in metastatic tumors and reduced Akt expression significantly inhibits metastasis in highly metastatic cancer cells [42]. We showed that MCME inhibited the Akt expression in CL1 cells. The ability of MCME to inhibit the prosurvival PI3K/Akt pathway in CL1 cells was extended from the downregulated Src and/or FAK and led to its anticancer activity. In addition, one of the essential functions of Akt is the phosphorylation of GSK-3*β* [26]. GSK-3*β* has constitutive kinase activity which is significantly reduced by phosphorylation at Ser9 [43]. Decrease of activated GSK-3*β* in MCME-treated CL1 cells indicated that the inhibited migration and invasion were mediated by the inactivation of Src, FAK, and the downstream GSK-3*β* through the PI3K/Akt/GSK-3*β* pathway. 

The Wnt signaling pathways play an important role in development and metastasis [44]. Wnt proteins bind to receptors belonging to the frizzled (Fz) family and activate an intracellular cascade that involves the inactivation of GSK-3*β*, ultimately resulting in the stabilization and nuclear translocation of cytosolic *β*-catenin [45, 46]. Nuclear *β*-catenin then interacts with various transcription factors, or targets to Wnt-regulated genes such as MMP-2 and MMP-9, to cause cellular proliferation or metastasis [38, 45, 47]. In the absence of a Wnt signal, *β*-catenin is degraded through GSK-3*β*-dependent or -independent pathways [47]. Since dysregulation of Wnt/*β*-catenin signaling pathway that leads to uncontrolled tumor cell proliferation, metastasis, and resistance to apoptosis has been reported in lung cancer [48, 49], Wnt and its downstream targets, GSK-3*β*, *β*-catenin, and MMPs, were included to study the effects of MCME on CL1 cells. Although GSK-3*β* was inactivated with an increased expression of Wnt-2 in MCME-treated CL1 cells, the expression of *β*-catenin was decreased. The downregulated *β*-catenin in MCME-treated CL1 cells accompanied by the declined expression of MMP-2 and MMP-9 indicated that degradation of cytosolic *β*-catenin was mediated by GSK-3*β*-independent pathways. There are multiple signaling mediators besides *β*-catenin whose degradation is specifically regulated by the Wnt/GSK-3*β* pathway [50]. In addition, a number of *β*-catenin-independent Wnt signaling pathways were identified to illustrate that not all functions of Wnt signaling can be attributed to canonical *β*-catenin-mediated transcription regulation [51–53]. Our results demonstrated that the increased expression of Wnt-2 was not tightly correlated with the expression of *β*-catenin and MMPs in MCME-treated CL1 cells. Moreover, inhibition of Src activity in MCME-treated CL1 cells attenuated the FAK-induced activation of GSK-3*β* and suppressed the expression of MMPs and cell motility. Accordingly, MCME, although not fully elucidated, may suppress lung adenocarcinoma cell invasion by inhibiting the expression of MMPs through the regulation of Src, FAK, PI3K/Akt, GSK-3*β*, and *β*-catenin. 

In this study, we investigated the protein levels of *β*-catenin, cell invasion markers including MMP2 and MMP9, and cell proliferation indicators including PI3K, Akt, and GSK-3*β* in CL1 cells with distinct metastatic capabilities. A remarkable different expression of Akt, *β*-catenin, and MMPs as well as the activation of GSK-3*β* was found in MCME-treated CL1-0 and CL1-5 cells in the earlier time. The decreased phosphorylation of Src and FAK in cells treated with MCME, at concentrations without significant drops in cell viability, demonstrated Src was not only involved in MCME-mediated antimetastatic effect but the tolerance to MCME-induced cytotoxicity. In addition, the significant increase of Wnt-2 in MCME-treated CL1-5 cells implies a process other than *β*-catenin for the survival regulation by its downstream targets. Further study of these Wnt/GSK-3*β* signaling pathways and their target proteins should provide valuable insights into the mechanism and regulation of Wnt and GSK-3*β* signaling pathways in MCME-induced cytotoxicity.

## 5. Conclusion

**Figure 6:**
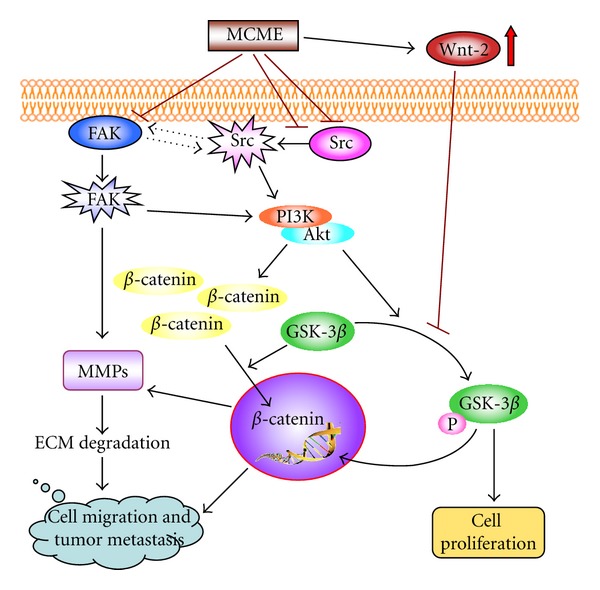
Proposed mechanism for MCME-mediated inhibition against migration of CL1 cells. The effect of MCME is mediated by both Src/FAK and Wnt signaling pathways. MCME reduces the expression and activation of Src and FAK, leading to the decreased expression of Akt which regulates the phosphorylation of GSK-3*β*. MCME-induced inactivation of GSK-3*β* is also regulated by the increased Wnt-2 after treatment. Declined expressions of *β*-catenin and MMPs demonstrate that antimetastatic activity of MCME-treated CL1 cells is mainly mediated through Src/FAK/Akt, but not Wnt-2/GSK-3*β*, pathway to inhibit *β*-catenin from entering the nucleus. In conclusion, Src, FAK, PI3K/Akt, and *β*-catenin are involved in the critical pathway to inhibit the metastasis through the downregulation of MMPs on MCME-treated CL1 cells.

The decreased expression and phosphorylation of Src, FAK, and the downstream regulators, PI3K/Akt and MMPs, in cells treated with MCME, is the critical event leading to cell death and antimetastasis in CL1 cells. Cellular exposure to MCME also eventually leads to GSK-3*β* inactivation. This proposed model of MCME-induced cell death and antimietastasis in lung adenocarcinoma CL1 cells is shown in Figure 6. This is the first paper to propose a sequence of events for MCME-induced cell death and metastatic suppression in human lung adenocarcinoma CL1 cells with different migration and invasion ability. Our data demonstrated that Src, FAK, and Wnt-2 are the upstream regulators in modulating cell proliferation and/or migration, leading to the inactivation of GSK-3*β* and/or subsequent decrease of *β*-catenin and MMPs in MCME-treated CL1 cells. Due to the anti-invasive effect at concentrations without significant cytotoxicity, CL1-5 cells were thought to be more tolerant to MCME. These results may lead to further studies to evaluate the possibility of MCME to be used as the dietary supplement on reducing human lung cancer tumorigenesis and/or progression.

## Supplementary Material

Momordica charantia-inhibited angiogenesis in a xenograft tumor model by breast cancer cells.
