# Supplementary material for: Antimigratory Effects of the Methanol Extract from Momordica charantia on Human Lung Adenocarcinoma CL1 Cells
Source: Evid Based Complement Alternat Med. 2012 Dec 18;2012:819632. doi: 10.1155/2012/819632 (PMC3535856; doi:10.1155/2012/819632)
Supplement: Supplementary file 1 — Momordica charantia-inhibited angiogenesis in a xenograft tumor model by breast cancer cells. [file 819632.f1.docx]

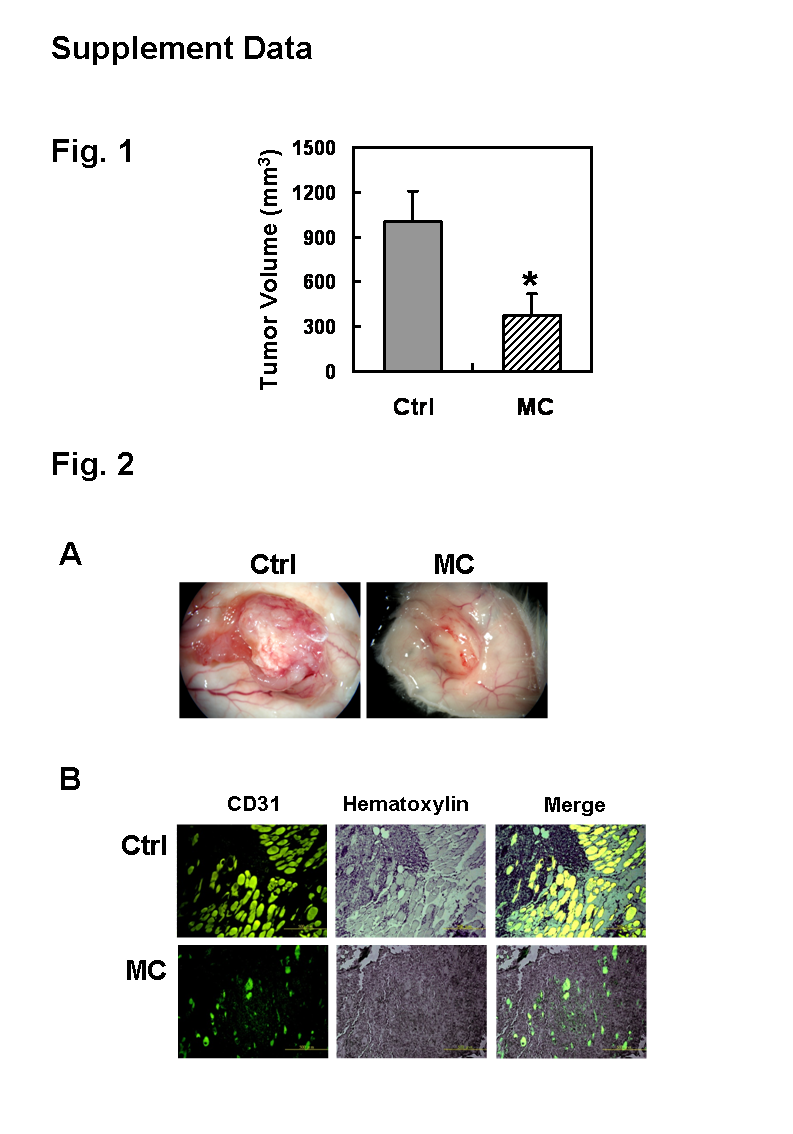


**Supplementary Figure Legends**

**Figure 1. *M. charantia*-inhibited tumor growth in the subcutaneous mouse tumor model.**

Mice were obtained from the Laboratory Animal Center, Tzu-Chi University, Taiwan. All animal experiments were conducted according to the ethical guidelines for the uses of animals and performed in compliance with the policies and procedure of the Institutional Animal Care and Use Committee of Tzu-Chi Universtiy. For the anti-tumor assay in SCID mice, animals, at least eight for each group, were given 1% (w/w) ethanol extract of *M. charantia* in their diet during the three-week experimental period, one week after MDA-MB-231 breast cancer cells (1×10^6^/animal) were inoculated into the right flank region. At the end of experiment, mice were sacrificed and tumors were excised carefully for measuring the tumor volume by using a Vernier caliper. Tumor volume was calculated using the ellipsoid volume formula: volume (mm^3^)=π/6(length)×(width)×(height). Starting from day 7 after inoculation by MDA-MB-231 cells, tumor growth in *M. charantia*-treated mice was significantly inhibited as compared with that of untreated mice (* indicates *P* < 0.05).

**Figure 2. Anti-angiogenesis of *M. charantia* on tumor-bearing mice inoculated by MDA-MB-231 cells.**

Mice were continuously treated with 1% (w/w) ethanol extract of *M. charantia* for three weeks. Tumors separated from mice inoculated subcutaneously with MDA-MB-231 cells were evaluated after treatments **(Figure 2A)**. Tumor of untreated mice was shown to be embedded with more vessels. Besides, tumor compassed by pink muscular tissue and vascular network in control mice indicated the invasion ability of the subcutaneous mouse tumor which could be inhibited by *M. charantia*. To further examine the anti-angiogenic effects of *M. charantia*, the density of intratumoral microvessels was analyzed by immunohistochemistry. The tumor mass was fixed with neutral buffer formalin, embedded with paraffin, cut into 5-μm thick sections with a microtome, and stained with hematoxylin or incubated with anti-CD31 (Platelet endothelial cell adhesion molecule-1, PECAM-1) antibody (GeneTex, Taiwan) for histopathological or microvascular evaluation. Even though there are still small vessels, tumors of mice treated with *M. charantia* displayed fewer vessels of luminal size than control tumors **(Figure 2B)**. Furthermore, CD31 staining revealed an aberrant morphology characterized by loosely distributed or absent endothelial cells in tumors of mice treated with *M. charantia*, suggesting that *M. charantia* inhibited tumor invasion as well as angiogenesis in mice inoculated subcutaneously by MDA-MB-231 cells.
